# Insights for speed management among Iranian drivers: a social marketing formative research study

**DOI:** 10.5249/jivr.v14i3.1690

**Published:** 2022-07

**Authors:** Mohsen Shams, Mostafa Maleki, Sedigheh Shariatinia, Afsaneh Omidimorad, Hormoz Zakeri, Mohsen Fallah Zavareh, Christoph Hamelmann, Ray Shuey, Mansour Ranjbar

**Affiliations:** ^*a*^ Department of Health Education and Promotion, School of Health, Yasuj University of Medical Sciences, Yasuj, Iran.; ^*b*^ Iranian Social Marketing Association, Yasuj, Iran.; ^*c*^ Department of Health Education and Promotion, School of Health, Tehran University of Medical Sciences, Tehran, Iran.; ^*d*^ Department of Health Education and Promotion, School of Health, Isfahan University of Medical Sciences, Isfahan, Iran.; ^*e*^ Department of Public Health, School of Health, University of Eastern Finland, Joensuu, Finland.; ^*f*^ Transportation Regulatory and Safety Center, National Road Safety Commission, Ministry of Roads & Urban Devel-opment, Tehran, Iran.; ^*g*^ Department of Civil Engineering, Engineering Faculty, Kharazmi University, Tehran, Iran.; ^*h*^ World Health Organization Representative in Iran, Tehran, Iran.; ^*i*^ Strategic Safety Solutions, Melbourne, Australia.; ^*j*^ World Health Organization Country Office, Tehran, Iran.

**Keywords:** Campaign, Social Marketing, Speed Management, Law Enforcement

## Abstract

**Background:**

Road traffic crashes are among the leading causes of death and disability in the world, particularly in low and middle-income countries. This study aimed at to conduct a social marketing formative research to inform the development of a campaign to manage driving speed on the intercity roads of Iran.

**Methods:**

To carry out the social marketing formative research, a qualitative and quantitative study were conducted. Also, a literature review of the speed management strategies was carried out and an analysis of traffic accident data and speeding violations was performed in selected provinces during 2019 and 2020.

**Results:**

Based on the findings of the qualitative study, perceived sense of lack of speed control, poor monitoring system, and law enforcement are the main reasons drivers exceed the speed limit. They mostly suggest using punitive levers and more stringent law enforcement practices for speed management. Literature reviews also confirm that to successfully develop an effective speed management plan a set of measures should be implemented together, including road safety engineering, raising awareness, social marketing strategies, and finally strict law enforcement. The overriding findings of the formative research revealed that to persuade Iranian drivers to respect the speed limit, the messages of the campaign should focus on strict law enforcement in the selected corridors.

**Conclusions:**

In the minds of the Iranian audience, strict law enforcement is of paramount importance for a speed management strategy to work; hence it should be taken into consideration when tailoring the campaign messages. From the findings of the present study, it can be concluded that to manage speed on intercity routes in Iran, a social marketing campaign is needed to encourage compliance with speed limits.

## Introduction

Road traffic crashes (RTCs) are among the leading causes of death and disability in the world, particularly in low and middle-income countries (LMICs).^1^ According to a report by the World Health Organization (WHO) in 2018, the number of people who have lost their lives in deadly road crashes has increased to 1.35 million. This is to say, every 24 seconds one person dies in a road crash.^2^ Approximately 63% of vehicles are in LMICs, yet 93% of deadly accidents happen in LMICs^3^ As the world population grows and with more use of motor vehicles, particularly in LMICs, it seems that the rate of deaths and injuries caused by road crashes in LMICs will rise in the coming years. This is a problem with proven solutions.

RTCs are a multifaceted problem, which can be broken into road-related factors, vehicle-related factors, and human factors.^4,5^ There are two approaches to avoiding RTCs: the traditional approach and the systemic approach. In the traditional approach, human error is thought to be the greatest contributor to fatal crashes. Therefore, the main focus of this approach has been to change the behavior of drivers, reduce human error and thereby prevent injury crashes.^6,7^ However, the systemic approach tries to prevent deadly crashes from happening by building and managing a safe system in which responsibility falls on the shoulders of both drivers, road designers, and managers.^8^ The Safe System Approach (SSA) has been promoted by the WHO as an effective measure to improve road safety and has been adopted in many developed countries.^9^ The SSA reframes the way in which road safety is viewed and managed. The SSA aims to eliminate fatal and serious injuries for all road users.^10^ Therefore, since safe speed acts as one of the main pillars of the SSA it should be specifically administered for each road, in such a way that crashes do not lead to death or severe injury.^11^

Data show speeding amplifies both the number and severity of crashes; hence speeding plays a significant role in more than one-third of deadly road crashes.^12^ The death toll caused by road traffic accidents in the WHO Eastern Mediterranean Region countries has risen from 17.9 per 100,000 in 2013 to 18 per 100,000 in 2016, and speeding has played a major role in many of these crashes and deaths.^1^

Driving over the speed limit is the biggest road safety problem in many countries, particularly in LMICs, including Iran.^5,13,14^ It is estimated that if effective speed management strategies in Iran were successfully implemented, and the driving speed across the country's road network fell by 10 km/h, the number of deadly road crashes would decrease by 40%.^15^

The speed management pilot project, based on the SSA and result-oriented management, will be implemented in three Iranian provinces: Khorasan Razavi, Isfahan, and Markazi. The present study is a part of the speed management pilot project, which was carried out with the support and advice of the Eastern Mediterranean Regional Office (EMRO) of the WHO.

In this project, the social marketing (SM) approach acts as a theoretical basis for designing the speed management intervention based on the SSA. SM, along with education and enforcement, is considered as the three main ways to change behavior. SM is at the forefront of audience-centered behavioral models, and its main objective is applying commercial marketing to shape the voluntary behaviors of target groups to boost their well-being and social welfare. In this approach, participants’ desires and needs are assessed, and then interventions are designed and implemented to decrease barriers or to reinforce the benefits of an idea, a behavior, or a social action to promote them.^16^ In the field of health, SM has been further defined as the customer-oriented use of commercial marketing principles and techniques to develop programs, interventions, and evaluations aimed to change health behaviors.^17^

The present study was conducted based on Social Marketing Assessment and Response Tool (SMART). This model has seven stages, in this study, three stages of formative research including audience analysis, market analysis and channel analysis were used. In SM formative research, a comprehensive analysis of behaviors is conducted for providing a comprehensive picture of the current patterns and trends of current audience behaviors.^18^ Examining the current behaviors of the target group and the relevant factors shaping them can accordingly help planners and managers have a clear picture of the target group and their problems to design and implement tailored intervention programs.

According to the exchange theory in SM , if certain measures are adopted to change drivers’ perception of the benefits of speeding, in such a way that drivers think of speeding as a costly behavior, they would be more likely to refrain from driving at illegal and unsafe speeds.^19^ Therefore, by listening to drivers who violate legal speed limits and collecting their ideas, one can understand decision-making processes about complying with speed limits. The results of this research help the behavioral intervention designers to understand how best to persuade drivers to voluntarily avoid speeding.

In many countries of the world, SM has been considered as the basis of addressing driving problems, such as decreasing risky driving behaviors,^20^ taxi drivers' views on risky driving behavior,^21^ reducing aggressive driving, reducing alcohol-impaired driving crashes,^22^ the "Drugged Driving Kills project: Why Drive High?”^23^

It seems that a majority of the drivers in Iran drive too fast, despite having enough information about the authorized speed and the consequences of speeding, as well as despite having the skill to drive without violating the speed limit.^24,25^ Moreover, despite having passed training sessions, these drivers do not respect the speed limit. Given the fact that driving at the speed limit does not require special skills and drivers can easily adhere to these limits instead of exceeding them, educational interventions are not particularly effective in encouraging drivers to respect the speed limitations. This study aimed at to conduct a SM formative research to inform the development of a campaign to manage driving speed on the intercity roads of Iran.

## Methods 

This article reports the application of the SM formative research to obtaining ideas for speed management intervention design. To do this, the following steps were carried out: a qualitative study, a cross-sectional survey, and an analysis of traffic crash data and speed violations in selected provinces. Besides, worldwide intervention strategies were reviewed to draw on other countries' best experiences. 

**The qualitative study**

***Study design***

The directional content analysis approach was used to conduct a qualitative study. This method is usually classified based on the theory-based inductive method, and its differences with other methods are based on the role of theory in them.^26^ A qualitative study was conducted based on the SM approach and its core concepts, particularly the SM mix namely product, price, place and promotion (4 Ps).^16^ The 4 Ps of marketing are the key factors that are involved in SM interventions. Marketing mix is a set of controllable tools that, by combining them, it will be possible to respond to the target market and audience group.^17^

The purpose of the qualitative study was to obtain the opinions of the interviewees as to why drivers violate legal speed limits, the obstacles to respecting speed limits, appropriate methods to persuade drivers to comply with the speed limits, effective communication channels, and suitable places to convey campaign messages to drivers. 

***Participants and Data collection***

In each of the pilot provinces, two officials from the Department of Highways and Road Transport, two officials from the Traffic Police, and two members of non-governmental organizations working in the field of road safety answered questions in the form of semi-structured-in-depth telephone interviews. In addition to interviewing these individuals, telephone interviews were conducted with selected drivers in the target provinces. In total, in-depth interviews were administered with 15 experts in the field of speed management and eight selected drivers in the target provinces. These people were selected purposefully. Department of Highways and Road Transport and Traffic Police in each provinces introduces key informant people. Telephone interviews were held by the second author of the article. The basis of data collection in qualitative studies is to achieve data saturation.^27^ Individual interview questions were tailored with an emphasis on the marketing mix. These questions include:

1) What is your general opinion about the current state of driving speed in the country? 

2) What factors do you think to encourage drivers to exceed the speed limits? 

3) If drivers in Iran wanted to follow the speed limits, what do you think would prevent them?

4) How do you think drivers can be persuaded to adhere to the speed limits? 

5) In your opinion, what options are available to communicate with drivers and leave an impact on their driving habits? 

6) Where do you think are the most suitable places to make an influence on drivers? 

***Data analysis***

Directional qualitative content analysis was applied to categorize the interviewees' answers to each question.^28^ Data analysis performed simultaneously with data collection and carried out by using directed content analysis in three phases, namely preparation, organization, and reporting. After each telephone interview, the audio file was transcribed by the interviewer. The collected data were analyzed using MAXQDA software version 10. Two Researchers examined the qualitative data separately and assigned their preferred codes to the participants' responses at the sentence level. After the coding was completed, the two researchers reviewed each other's codes and resolved issues of disagreement through discussion. In cases where no agreement was reached, a third party assisted.

**The analysis of traffic accident data and speeding violations in the selected provinces**

The information available in the databases of institutions dealing with traffic crashes and speeding violations in selected provinces of the country were consulted to determine the specific target audience and intercity corridors suitable for the administration of the pilot study in the campaign designing. Having granted the consent of the country's officials and officials in the selected regions, the required data were provided to the research team. 

**Cross-sectional study**

The cross-sectional study was done to a deeper understanding of the factors affecting speeding among drivers in the selected provinces. To do this study, we applied theory of planned behavior (TPB). The TPB is a psychological theory that links beliefs to behavior. The theory maintains that three core components, namely, attitude, subjective norms, and perceived behavioral control, together shape an individual's behavioral intentions.^29^ According to the TPB, the behavioral intention is the most proximal determinant of human social behavior.^30^ This theory has been applied to driving behaviors such as aggressive driving,^31^ texting while driving,^32^ using mobile phones while driving,^30^ and driving while drunk.^33^

Data was collected by using TPB questionnaire. The questionnaire was developed by Boissin and et al. to determine the factors affecting speeding among new generation of Omani drivers.^34^ The questionnaire was translated and localized by a member of the research team to be used among drivers in Iran. After this, the questionnaire was compiled into an electronic version, and drivers were asked to answer it using a link. Finally, the quantitative survey was completed by 164 drivers in the selected provinces. Data obtained from the cross-sectional survey were analyzed using SPSS version 16.

**Speed management literature review**

A global literature review of the speed management strategies was carried out to benefit from successful experiences in other countries. With this objective in mind, search operations by using the keywords "driving speed management" and "reducing driving speed" were performed on Google Scholar and PubMed databases, as well as Persian data base Magiran, SID, and IranMedex. All articles published in English or Persian from 2000 to 2020 were searched in these databases. Papers that aimed at driving speed management were selected. 

**Ethical Considerations**

At all stages of the study, participants were given a full explanation about the objectives of the study and voluntary participation. After that their informed consent was obtained and data were collected. Participants were assured that their information would remain confidential. 

## Results

This study aimed to conduct SM formative research to inform the development of SM campaign to manage driving speed on intercity routes in a selected number of provinces in Iran. In the following sections, the findings of each step of the study are presented. 

**The findings of the qualitative study**

In this stage, 23 people, including drivers and people working in the field in the three target provinces were interviewed. Almost all the interviewees stated that most drivers do not respect the speed limit on intercity routes. Respondents' answers to the question of why drivers exceed the authorized speed limits are summarized in Table 1. Moreover, their suggestions for respecting the speed limit on intercity routes are presented in Table 2. 

**Table 1:** Drivers’ reasons for speeding and number of codes extracted for each one.

The core concepts		Number of Codes
Reasons for speed violation	Poor monitoring system and a perceived belief that there is not a speed control mechanism in place. (insufficient number of speed cameras, broken cameras, poor monitoring of speed violations, delay in receiving speeding fines)	44
	Ineffective speed management training at the time of obtaining a driving certificate (insufficient effectiveness of speed management training, driver's poor skills in speed management, lack of additional training courses, inconspicuous speeding consequences for drivers while in the training stage, unfamiliarity with the authorized speed limit)	31
	Negligence in addressing the crimes related to speed violations	19
	Being in a state of hurry	19
	Speeding fines are not deterring	16
	Improper location of speed signs	11
	Momentary effectiveness of point cameras in preventing speed violations	9
	Not observing the speed limit	9
	Corruption in the implementation of the law	7
	Lack of signs and warning signs	7
	The excitement of driving at high speed	6
	Driver distraction	5
	The natural human desire to speed	4

**Table 2:** Interviewees' suggestions for speed management and the number of codes extracted for each one.

The core concepts		Number of Codes
Suggestions for speed management strategies	Use of punitive levers (temporary ban on driving, higher speeding fines, and more expensive insurance premiums for speeding offenders)	30
	Smart monitoring system	28
	Immediate and decisive confrontation with speeding offenders (sending a prompt notification to the speeding driver and dealing with speeding offenders without negligence)	26
	Highlighting the consequences of speeding (making documentaries about people involved in accidents and their families, warning the driver about the speed limit, informing drivers about the consequences of speeding, installing warning signs)	24
	Raising Awareness	17
	Encouraging good driving habits	15
	Studying drivers' speed management techniques in hazardous situations	11
	Teaching healthy driving practices at schools	9
	Establishment of welfare service complexes along the roads	3

The respondents believed that suitable places for the implementation of the speed management project were: gas stations, roadside welfare complexes, toll booths, roadside shopping malls, road police stations, highways and road transport stations, along the roads, city exits, and road stations in summer and new year travels. When asked about the proper communication channels for conveying campaign messages to drivers, the respondents mentioned television and radio with an emphasis on television, billboards, posters and brochures, cyberspace, and the Internet, respectively.

Almost all experts and drivers believe that the monitoring system and law enforcement are not properly administered in Iran. Drivers feel that no one is monitoring them on the road. They also believe that in terms of being subjected to punishment there is no difference between a driver who respects the speed limit and a driver who does not. In general, the findings show that speeding violations in Iran are not met with strict law enforcement.

**Findings of data analysis regarding traffic accidents and speed violations**

The analysis of data regarding the traffic accidents and speed violations on intercity routes in selected provinces revealed that the following routes are suitable for the project administration: Isfahan-Morche Khort-Meymeh-Delijan route and Najafabad-Tiran-Daran route in Isfahan province, Kahak-Sabzevar-Neishabour route, and Chenaran-Qoochan-Farooj route in Khorasan Razavi province, Saveh-Tehran freeway route and Arak-Salafchegan route in Markazi province. The analysis of these data also indicated that a suitable target group for the implementation of the pilot project includes male drivers aged between 20 to 50 who own a private car. 

**Results of the literature review of speed management strategies**

A total of 18 studies was were reviewed. Results of the literature review show that making a significant change in the average driving speed is not feasible without conducting long-term and continuous intervention measures.^35,36^ Successful and effective speed management practices depend on strong political support, regular and systematic monitoring of road safety levels, and continuous evaluation of executive actions.^14^ Furthermore, any speed management intervention should be implemented based on accurate assessments of the current situation as well as available resources and technologies so that the plan remains active over time.^35^ Another important finding of the literature review was that intervention programs based on educational and SM, engineering, and law enforcement should be conducted in conjunction with each other.^37^ The literature recommends that, in LMICs, low-cost solutions to fit with existing resource limits and technology could be used.^5^ The next step after that is to design and implement educational awareness campaigns and SM programs with an emphasis on speed limits to inform drivers that exceeding speed limits is not wise behavior as they will face punishment and legal fines.^38^ Finally, active law enforcement should be administered for drivers who do not comply with the speed limits.^39^

Law enforcement can be seen in almost all strategies used for speed management. Also, the use of speed cameras is one of the most widely used methods in speed management programs. One notable finding was that when speed cameras are used as a speeding detection system, it is necessary to promptly inform drivers of their violations and a fine ticket is sent to them.^14,40,41^ Also, it is of paramount importance to follow up on delays in the payment of driving fines so that their preventive role is observed. Moreover, some studies show that removing speed cameras from a specific road leads to higher driving speeds on that route.^41^

Besides, the results of the literature review show that the use of different speed management strategies often depends on road characteristics and drivers' attitudes toward these strategies, as well as the economic and technological capabilities of the country.^42^ Therefore, speed management interventions should be designed in such a way that most drivers accept them. Speed management interventions also show that in designing the intervention programs, it must be clear why drivers do not adhere to the speed limits. In other words, designers of speed management interventions need to know the motivations of drivers exceed legal speed limits.

**The quantitative study findings**

The mean and standard deviation of the age of the respondents was 35/92 and 7 years, respectively.Table 3 presents the demographic characteristics of the drivers. According to drivers' self-reports, most drivers have been fined for speeding over the past two years. In general, the quantitative survey found that most drivers are fully aware of the consequences of speeding and accidents caused by driving too fast. More than 90% of them believe that adhering to the speed limits cuts down the number of crashes and injuries. However, almost 20% of drivers believed that the risk of being fined and prosecuted for speeding was very slim. Also, most drivers have a positive attitude towards following the speed limit (Table 4). However, results indicate that driving fast and not respecting the posted speed limits was common in younger drivers who are less than 30 years. Drivers who had a positive attitude and higher perceived behavioral control towards speeding were more likely to intend to engage in safer driving behaviors (p<0.001). 

**Table 3:** Demographic profile of respondents to quantitative survey questions.

	Demographic Variables	Number (Percentage)
**Education Level**	Under Diploma	(6.1) 10
	Diploma	22 (13.4)
	Academic Degree	132 (80.5)
**Is driving your job?**	Yes	27 (16.5)
	No	137 (83.5)
**What type of car do you usually drive? **	Personal Car	146 (89)
	Utility vehicle	1 (0.6)
	Taxi	0
	Bus	3 (1.8)
	Minibus	2 (1.2)
	Truck	12 (7.3)
**Approximately how many kilometers have you driven last week?**	Less than 100 kh/h	(32.9) 54
	Between 100 to 300 kh/h	45 (27.4)
	More than 300 kh/h	65 (39.6)
**Have you been prosecuted for any motoring offences in the last two years?**	Yes	110 (67.1)
	No	54 (32.9)
**For what traffic offences have you fined? **	Illegal speed	58 (50.9)
	speeding	9 (7.9)
	Not wearing seatbelt	16 (14)
	Using mobile phone	14 (12.3)
	Deviation to left	0
	Dangerous overtaking	0
	Condition of vehicle	4 (3.5)
	Document offences	3 (2.6)
	other	39 (34.2)

**Table 4:** Relative frequency of drivers’ agreement or disagreement with speed-related statements.

I perceive that respecting the speed limit will	number (percentage)				
	Strongly agree	agree	Neither	disagree	Strongly disagree
Reduce the probability/risk of being involved in road traffic crash.	87 (53)	69 (42.1)	2 (1.2)	3 (1.8)	3 (1.8)
Reduce the probability/risk of hurting someone else in road traffic crash.	87 (53)	70 (42.7)	4 (2.4)	1 (0.6)	2 (1.2)
Reduce the probability for being stopped by the police.	70 (42.7)	65 (39.6)	16 (9.8)	8 (4.9)	5 (3)
Increase my chances to survive road traffic crash.	90 (54.9)	68 (41.5)	1 (0.6)	4 (2.4)	1 (0.6)
Make it possible to stop in time to avoid road traffic crash.	02(62.2)	57 (34.8)	1 (0.6)	2 (1.2)	2 (1.2)
Causes less damage to the car in road traffic crash.	91 (55.5)	68 (41.5)	1 (0.6)	1 (0.6)	3 (1.8)

The items that always or most often reminded drivers to respect the speed limit were: traffic signs (70%), observing a crash scene (68%), speed detecting signs (65%), crashes involving family and friends (58%), family members (45%) and speeding fines (43%). Quantitative survey findings also showed that encountering a crash scene (68%), speed signs (66%), family members (66%), traffic signs (65%), and crashes involving relatives and friends (60%) are among the factors that always or oftentimes encourage drivers to drive at the speed limit. The factors that help drivers to respect the speed limit are speed cameras (77%), stationary police cars (76%), police patrol cars (70%), imperceptible speed monitoring system (60%), text messages containing police warnings (64%) and getting pulled over by the police (63%), respectively. 

**Insights for SM campaign development**

• Analysis of data on road accidents, drivers of vehicles involved in accidents, and speeding offenders determined that male drivers between 20 and 50 years of age who drive a private car are the best and most suitable target group for the project. Also, by determining two corridors in each of the three target provinces of the program, the location of the campaign was determined.

• Regarding the specific target group and the selected routes for the campaign, it should be noted that these drivers are traveling in the selected corridors for a short time and the intervention plan is designed according to these limitations. Drivers entering the selected corridors of the program will be informed about the differences between these routes and other routes and will be warned that in these routes, legal consequences of speeding violations are serious.

• Findings of the quantitative and qualitative studies also support that speed management plans in Iran ask for legal and regulatory measures to be strictly applied and drivers who do not follow the rules should face the consequences. Since law enforcement is highly prominent in the minds of the Iranian audience, it should address in the messages of the SM campaign. 

• To gain the attention of drivers, campaign messages should highlight the differences between selected corridors with other roads and paths. Also, for many drivers and experts, highlighting the legal and health consequences of speeding, particularly getting pulled over by the police and facing penalties, suffering injuries, and even death in a crash are effective reminders to respect the legal driving speed. For this purpose, four messages were specifically tailored to encourage drivers to respect the speed limit. The main contents of the messages are speed control by the fix and mobile cameras, stopping the offenders by the Police, heavy fines of offenders, and more mortality and morbidity. Therefore, without improving the law administration process and strictly monitoring it, one cannot expect any improvement to come from the SM campaign. Strict law enforcement by the police plays a key role in completing and advancing the speed management intervention plans based on the SSA.

• The SM campaign should influence the minds of drivers and encourage them to drive at the permitted speed. In this persuasion, the main focus of the program and its components is to highlight the health and legal consequences of driving at illegal speed. 

• The aim of the campaign is to improve the behavior of drivers and reduce the percentage of drivers who drive at illegal speeds. After preparing all the stages of the program, a two-week period can be used to inform and justify the drivers entering these selected routes. This opportunity is used to explain the program in general, how it is implemented, goals, and when the program will start, in order to provide a mental background for the implementation of the program.

• The success of the project depends on the appropriate support of legislators, policymakers and managers, as well as the participation of drivers. In order to create a commitment to support and implement this program, it is necessary to involve them.

## Discussion

The aim of the present study was to gain insights to guide intervention development. To do this, SM formative research was applied. According to opinions of interviewees, highlighting the consequences of speeding, especially being stopped by the police and being fined, getting into an accident and being injured in an accident, and even dying, are good reminders to respect the speed limit. Therefore, these items should be considered in designing the campaign. In many other studies, reminders have been used to comply with the speed limit. For example, speed limit flashing lights,^39^ signs indicating the speed of drivers,^42^ increasing the number of fixed and mobile speed control cameras^43^ have been used in different countries.

By determining two corridors in each of the three provinces and considering male drivers aged 20 to 50 who drive in these corridors, the specific target group of the social marketing campaign was formed. The designers of the intervention for speed management in Ghana also identified the areas where the most speed violations were committed.^43^ In Italy, a highway was selected for speed management intervention.^44^ In Tehran, in order to reduce dangerous driving behavior, the drivers of the two regions with the most violations were selected.^20^

The findings of quantitative and qualitative studies emphasize the need for legal and regulatory measures to identify drivers who drive fast and deal with them. In line with the findings of the present study, Gupta has also mentioned in designing social marketing campaign that a set of measures including consolidating legal action, seeking support for legislation, police training, and developing a SM campaign for speed management should be taken into consideration.^14^ In this regard, many intervention programs have used a set of legal and engineering measures combined with behavioral interventions.^37,41,42,45-47^ For example, in designing a public campaign called "Safe Speed", the focus was firstly on informing drivers of the dangers of speeding and the role of speed cameras. Later, drivers were informed that speed cameras have been installed in different areas.^40^ Another SM campaign, called Wipe Off 5, was designed to support the intensification and expansion of the police actions in controlling speed measures.^45^ In the speed management intervention plans in Canada, a series of legal and educational measures were taken to ensure drivers comply with the new speed limit.^42^

Results of the online survey indicated that Iranian young drivers often do not follow the speed limit and are interested in speeding. This finding in consistent with similar studies. In Australia, Oman, Turkey and most of other comprise young drivers drive too fast are the target group for many speed management interventions.^14,48,49^ Moreover, positive attitude and higher perceived behavioral control towards speeding were the important predictors of the intention of respect to the posted speed limit. In line with the results of a quantitative study, among Indonesian and Omani drivers, speed intention is directly influenced by attitude. Attitudinal messages were used in the design of speed management interventions in these two countries to reduce driving speed.^50,51^

This study must be viewed in light of limitations. The focus of the study was on speed management on intercity roads, and this issue should be taken into account when interpreting and generalizing the results. Also, the research team was supposed to make a field visit to the studied provinces, but it was not done due to the epidemic of Covid-19. The literature review was not systematic and restricted to the scientific-reviewed paper and Google Scholar and PubMed databases. However, with these limitations in mind, according to the results of the formative research, it can be recommended and concluded that male drivers aged 20 to 50 who drive their private cars were the most appropriate target group for the project. Moreover, by defining two corridors in each of the three target provinces, the audience of the SM program was formed. The SM approach intends to leave an impact on drivers and encourage them to drive at the speed limit. In this encouragement, the main focus of the program and its components is on highlighting law enforcement, as well as highlighting the health and legal consequences of speeding. 

**Acknowledgment**

Special thanks to Dr. Hala Sakr Ali, Violence, injuries and disabilities, (WHO), Regional Office for the Eastern Mediterranean, and Dr. Lori Mooren, Safety and Communications, Sydney, Australia. 
